# Glutathione-*S*-Transferases as Potential Targets for Modulation of Nitric Oxide-Mediated Vasodilation

**DOI:** 10.3390/biom12091292

**Published:** 2022-09-13

**Authors:** Tiffany M. Russell, Des R. Richardson

**Affiliations:** 1 Centre for Cancer Cell Biology and Drug Discovery, Griffith Institute for Drug Discovery, Griffith University, Brisbane 4111, Australia; 2 Department of Pathology and Biological Responses, Graduate School of Medicine, Nagoya University, Nagoya 466-8550, Japan

**Keywords:** glutathione-*S*-transferase, nitroglycerin, nitric oxide, vasodilation

## Abstract

Glutathione-*S*-transferases (GSTs) are highly promiscuous in terms of their interactions with multiple proteins, leading to various functions. In addition to their classical detoxification roles with multi-drug resistance-related protein-1 (MRP1), more recent studies have indicated the role of GSTs in cellular nitric oxide (NO) metabolism. Vasodilation is classically induced by NO through its interaction with soluble guanylate cyclase. The ability of GSTs to biotransform organic nitrates such as nitroglycerin for NO generation can markedly modulate vasodilation, with this effect being prevented by specific GST inhibitors. Recently, other structurally distinct pro-drugs that generate NO via GST-mediated catalysis have been developed as anti-cancer agents and also indicate the potential of GSTs as suitable targets for pharmaceutical development. Further studies investigating GST biochemistry could enhance our understanding of NO metabolism and lead to the generation of novel and innovative vasodilators for clinical use.

## 1. Introduction

Glutathione-*S*-transferases (GSTs) are a superfamily of phase II detoxification enzymes and ligandins ubiquitously expressed in most living organisms and account for 1% of cellular protein [1,2]. These enzymes are divided into seven classes (α, µ, π, σ, θ, ω, and ξ) that are characterized by sequence similarity and immunological cross-reactivity [3,4]. Cytosolic GSTs are further divided into 16 gene-independent classes distinguished by sequence homology, substrate specificity, inhibitor sensitivity, and immunological properties [3,4].

While GSTs are traditionally associated with detoxification mechanisms due to their ability to conjugate glutathione (GSH) to toxins for excretion, recent advances have explored the role of GSTs in NO metabolism [5,6,7,8,9,10]. Studies investigating the extensive role of NO in vascular reactivity have identified GSTs as targets for the biotransformation of organic nitrates, including nitroglycerin, that results in vasodilation [11,12,13,14]. Intriguingly, there are several existing relationships between GSTs and the regulation of NO metabolism, particularly examining macrophages and tumor cells [8,9,15,16]. This function is related to the rich chemistry of NO coordinating to iron [8,15,16,17,18,19,20] to form dinitrosyl-dithiol iron complexes (DNICs) that spontaneously form upon the interaction of iron, NO, GSH, or cysteine [6,10,21,22,23,24].

**Figure 1:**
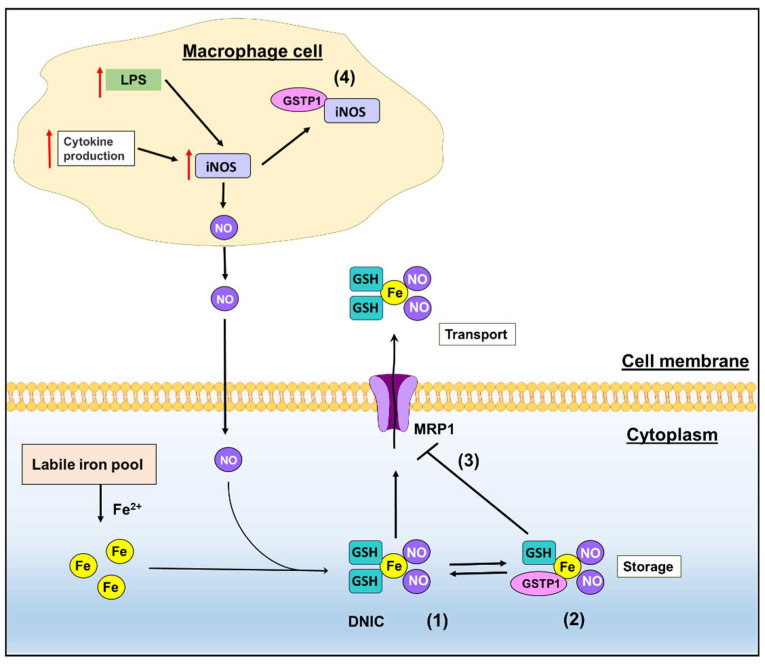
Schematic of the functions of GSTP1 in NO metabolism where: (**1**) NO binds to iron and GSH to generate small molecular weight dinitrosyl-dithiol iron complexes (DNICs); (**2**) DNICs then bind to GSTP1 to lead to a store of NO; (**3**) the binding of DNICs by GSTP1 prevents their transport out of the cell by MRP1; and (**4**) GSTP1 can also bind to inducible nitric oxide synthase (iNOS) that generates intracellular NO.

These relationships are mediated by: (1) the formation of DNICs [6,10,21,22,23,24]; (2) the direct binding of DNICs by GSTP1 to form a stable store of NO [8,9,15,16]; (3) the storage of DNICs by GSTP1 that then leads to a decrease in DNIC transport out of the cell by multi-drug resistance-related protein 1 (MRP1) [8,15]; and (4) the direct association of GSTP1 with inducible nitric oxide synthase (iNOS) to increase its degradation [5] (Figure 1). Overall, GSTP1 acts to bind and store NO, but also inhibits iNOS expression to suppress NO signaling.

## 2. GSTs and Emerging Roles in NO Metabolism

Studies by Cesareo and colleagues reported a crystal structure of a DNIC bound to GSTP1-1 [6]. This interaction with GSTP1-1 markedly increased the half-life of free NO from seconds to 8 h [6,7]. Further studies identified the binding of DNICs to other GST isotypes, namely GSTA1 and GSTM1, which were also able to increase the half-life of NO to approximately 4.5 h [6,7]. Although the function of these GST-DNIC complexes is not well understood, several studies have explored the role of GSTs in DNIC storage and the subsequent transport of DNICs out of cells via the GSH transporter, MRP1 [8,9,15,16].

Lok and colleagues proposed a model in which DNICs behave as a “common currency” for NO transport and storage via MRP1 and GSTP1, respectively, in breast cancer cells and also several macrophage models [8,15]. These studies were based on: previous investigations exploring the interaction of GSTs and DNICs [6]; that MRP1 could transport DNICs in tumor cells [25]; and that GSTs (GSTA1, GSTM1 and GSTP1) protect hepatocytes from the cytotoxic activity of NO [21]. In studies using MCF7 breast cancer cells, a significant decrease in NO-mediated iron release from cells by the GSH transporter, MRP1, was observed in GSTP1-overexpressing MCF7 cells [15]. It was demonstrated that this decreased transport of iron was due to the increased binding of DNICs to GSTP1, and the intrinsic storage of stable NO (as DNICs) by GSTP1 (Figure 1) [15].

Subsequent studies examining activated macrophages demonstrated that silencing Mrp1 resulted in an intracellular accumulation of DNICs, while silencing Gstp1 in these cells augmented the release of iron-59 out of the cell (as DNICs) [8]. Another intriguing GSTP1-NO interaction has been suggested by studies demonstrating the binding of GSTP1 to iNOS [5]. In this later study, GSTP1 was shown to directly interact with the oxygenase domain of iNOS through the GSTP1 G-site domain [5]. The interaction between GSTP1 and iNOS resulted in decreased iNOS dimer levels by the enhanced *S*-nitrosylation of iNOS and its ubiquitination, leading to reduced iNOS stability [5].

## 3. Nitric Oxide: A Hallmark Vasodilator

A hallmark function of NO is its ability to modulate signaling pathways, which occurs via the binding of NO to the heme prosthetic group of soluble guanylate cyclase (sGC) [26,27,28,29]. This NO-sGC interaction produces a heme–iron–nitrosyl complex that can activate the enzyme [22,29]. Activation of sGC results in the conversion of guanosine triphosphate (GMP) into the secondary messenger cyclic guanosine monophosphate (cGMP), which is central to myriad downstream processes, including vasodilation [30,31,32,33].

**Figure 2:**
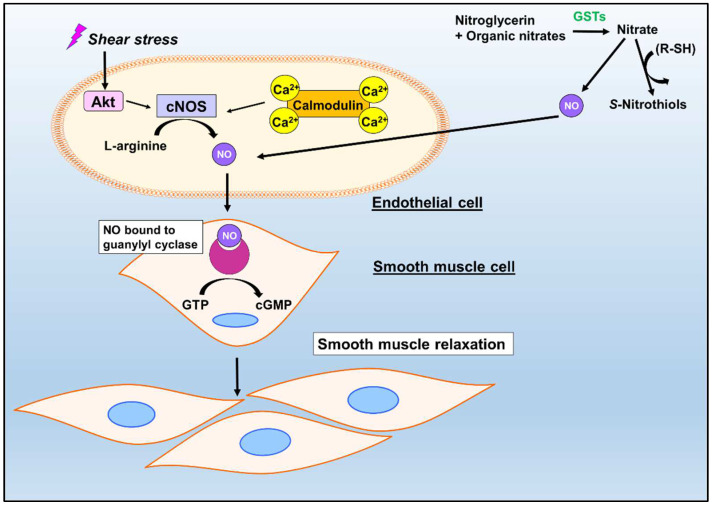
Schematic of the NO and potential GST-mediated regulation of vasorelaxation via sGC activation. Upon activation of constitutive nitric oxide synthases (cNOS; composed of endothelial and neuronal NOS) by calmodulin and calcium, cNOS can catalyze the conversion of L-arginine to L-citrulline to generate NO [37,38,39,40]. The production of NO can also result from the breakdown of organic nitrates, such as nitroglycerin [11,12,13,14]. NO facilitates the activation of sGC and the subsequent conversion of GTP to cGMP for vasodilation [37,38,39,40].

The function of NO in smooth muscle cell relaxation is well-established [34,35,36]. Endothelial NOS (eNOS) production is highly dependent on calcium and calmodulin (CaM) [37,38,39,40]. Increased Ca^2+^ levels enhance the affinity of CaM for eNOS, which promotes the conversion of L-arginine to L-citrulline and the production of NO (Figure 2) [37,38,39,40]. The activation of cGMP stimulates the activation of protein kinase G (PKG) and myosin phosphatase, which results in increased calcium release from intracellular stores, inducing smooth muscle relaxation (Figure 2) [40,41]. While no studies have explored the direct relationship between GSTs and NO in vasodilation, there have been multiple reports that indicate a potential link between GSTs and the denitration of vasodilators and organic nitrates for vasorelaxation [11,42,43,44,45,46,47]. These investigations are described below.

## 4. Biotransformation and Bioactivation via GSTs

### 4.1. Biotransformation of Organic Nitrates

Organic nitrates (R–ONO_2_) are efficacious pro-drugs that result in NO generation, which promotes vasodilation and decreases blood pressure [48,49,50,51,52]. These drugs have been widely utilized by patients for over a century, although their mechanisms of action are still not completely understood. Early observations regarding the biotransformation of organic nitrates demonstrated that GSH was required for the conversion of the organic nitrates, nitroglycerin and erythritol tetranitrate, into inorganic nitrate (ONO^−^) and oxidized GSH (GSSG) [53].

**Figure 3:**
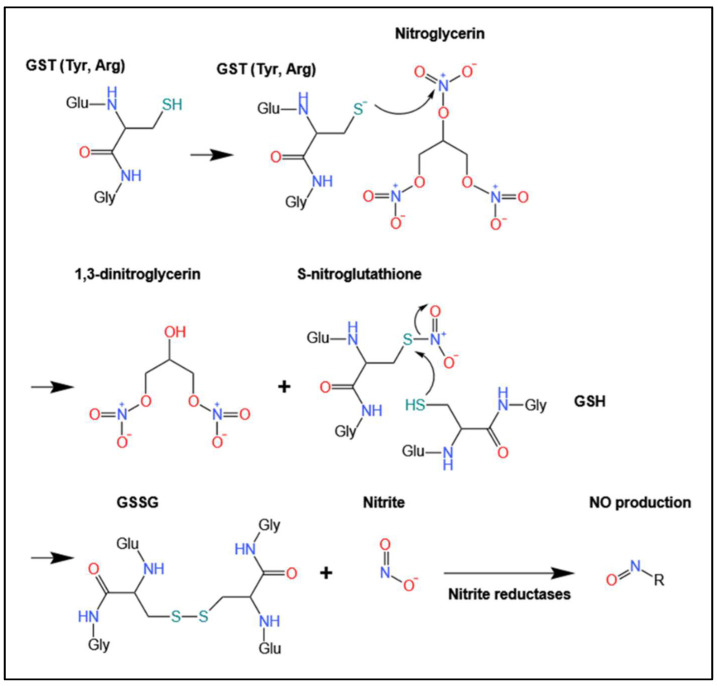
Schematic of the proposed mechanism for the biotransformation of nitroglycerin to form GSSG and nitrate via a mechanism mediated by the binding of GSH to GST. This scheme has been modified from [56].

It was identified by Jakoby and colleagues [54] that GSTs catalyze the biotransformation of nitroglycerin, erythritol tetranitrate, isosorbide dinitrate (ISDN), and ethylene glycol dinitrate, to nitrite and GSSG. The mechanism of this GST-catalyzed reaction is thought to involve the nucleophilic attack of the sulfhydryl group of GSH (bound to GST) onto one of the electrophilic nitro groups of nitroglycerin (Figure 3) [55,56]. This reaction produces 1,3-dinitroglycerin and *S*-nitroglutathione (GSNO_2_), the latter being an unstable intermediate (Figure 3) [55,56]. It is suggested that GSNO_2_ then non-enzymatically reacts with another GSH molecule to generate GSSG, resulting in nitrite release [55,56]. The nitrite is then converted to NO via nitrite reductases (Figure 3) [57].

Notably, the cooperation of tyrosine and arginine residues in GSTs has been proposed to be responsible for the deprotonation of the SH group within GST-bound GSH (Figure 3) [58]. Direct proof of the GST-catalyzed generation of GSNO_2_ from pharmacological organic nitrites, such as nitroglycerin, has yet to be established. However, studies using GST inhibitors demonstrate a direct correlation between GSTs, the organic nitrate-mediated release of NO, and subsequent vasorelaxation [42,43,44,45,46,47].

### 4.2. Biotransformation of Other Pro-NO Drugs by GSTs

**Figure 4:**
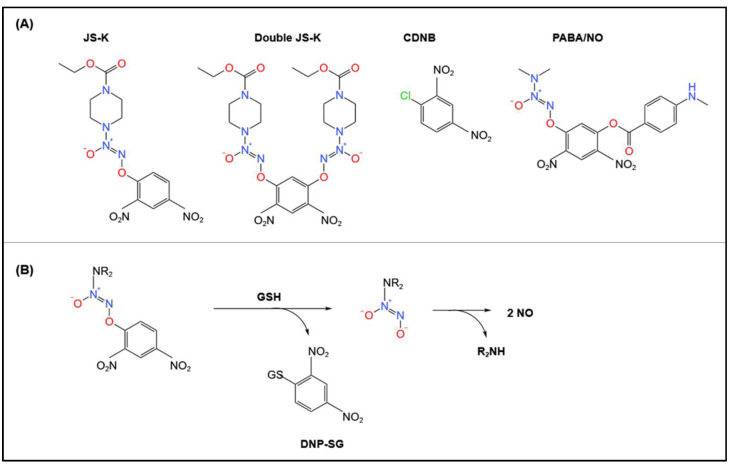
Pro-drugs metabolized by GSTs. (**A**) Line drawings of the chemical structures of common pro-NO drugs metabolized by GSTs. (**B**) Schematic describing the general mechanism of pro-NO drug biotransformation by GSTs.

More recently, other NO-generating agents, such as the diazeniumdiolate pro-NO drugs that are activated by GSH via GSTs, have been studied in terms of developing novel anti-cancer drugs [59,60,61,62,63,64,65]. These compounds take selective advantage of the elevated GST levels within tumor cells to induce their anti-cancer activity [59,60,61,62,63,64,65]. However, GST-mediated catalysis of NO from the pro-drug, O^2^-(2,4-dinitrophenyl) 1-[(4-ethoxycarbonyl)piperazin-1-yl]diazen-1-ium-1,2-diolate (JS-K), has been demonstrated to promote vasodilation, which limits its use for cancer treatment [59,61]. While the structures of these compounds (Figure 4A) are dissimilar to the organic nitrates mentioned above (Figure 3), the mechanism of their GST-mediated biotransformation leading to NO is similar (Figure 4B).

Common pro-NO drugs include JS-K, 1-chloro-2,4-dinitrobenzene (CDNB), and O^2^-{2,4-dinitro-5-[4-(*N*-methylamino)benzoyloxy]phenyl} 1-(*N*,*N*-dimethylamino)diazen-1-ium-1,2-diolate (PABA/NO) (Figure 4A). The general mechanism for the biotransformation of these agents involves a GST-catalyzed nucleophilic aromatic substitution by GSH leading to the common product, *S*-2,4-dinitrophenylglutathione (DNP-SG) (Figure 4B). The diazeniumdiolate anion product then spontaneously decomposes to generate NO.

Interestingly, a second-generation JS-K analog, “double JS-K”, has been developed to generate higher concentrations of NO (4 mol NO/mol of compound) and is similarly metabolized by GSTs [66]. Although there are reports that certain pro-NO drugs react with GSH in the absence of GSTs [59,64], enhanced NO generation from JS-K has been observed with increased cellular GST levels [61]. These pro-NO drugs are of interest, as the ability of GSTs to metabolize these agents, including organic nitrates, may be relevant to developing pharmaceuticals targeted towards NO production, such as new vasodilators.

## 5. GST Inhibitors Prevent Organic Nitrate-Induced Vasodilation

Several GST isoforms have been characterized in vascular smooth muscle cells, with GSTM1 demonstrating metabolic activity towards organic nitrates [12,13]. An investigation by Yeates and colleagues examined the role of GST inhibitors such as sulphobromophthalein on the spasmolytic activity of nitroglycerin and demonstrated that its dose–activity curve was displaced to the right [43]. It was shown in this study that GST activity within aortic homogenates was suppressed by sulphobromophthalein and that incubation of aortic strips with this inhibitor decreased relaxation induced by the NO-generating compound, *S-*nitroso-*N*-acetyl-penicillamine (SNAP), versus the control [43].

This later study was the first to provide evidence of the role of GSTs in the in vivo activation of organic nitrates. Moreover, these authors proposed a mechanism for the stepwise activation of nitroglycerin and other organic nitrates to *S*-nitrosoglutathione and NO for the relaxation of the aorta [43]. The impact of sulphobromophthalein on nitroglycerin metabolism has also been observed in several other studies [12,45,67].

The effects of sulphobromophthalein and another GST inhibitor, ethacrynic acid, on nitroglycerin metabolism were investigated in rabbit aortic strips [42]. Precontraction of the strips with phenylephrine followed by relaxation with nitroglycerin in the presence of ethacrynic acid resulted in a 32% inhibition of nitroglycerin-induced relaxation [42]. Unlike the previous report of Yeates and associates [43], incubation with sulphobromophthalein did not significantly decrease nitroglycerin activity [42]. To observe the metabolism of nitroglycerin, the dinitrate metabolite of this vasodilator, namely 1,3-dinitroglycerin (Figure 3), was measured within rabbit aortic tissue and was decreased in response to ethacrynic acid [42]. A significant correlation was observed between the ethacrynic acid-induced reduction in nitroglycerin activity and its inhibited metabolism [42]. Furthermore, dose–response curves revealed that ethacrynic acid suppressed nitroglycerin-induced relaxation [42].

The impact of ethacrynic acid on nitroglycerin metabolism was also investigated by Kenkare and Benet in studies using rabbit aortic strips [68]. It was demonstrated that nitroglycerin-induced relaxation and the increased cGMP levels were markedly decreased when strips were pretreated with ethacrynic acid [68]. Collectively, these studies demonstrate that GSTs, which are inhibited by ethacrynic acid, may be crucial in the vascular activation of nitroglycerin that is involved in vasorelaxation.

### Impact of GST Inhibitors on the Half-Life of Nitroglycerin

In additional investigations, Benet and colleagues investigated the role of GSTs in 1,3-dinitroglycerin generation from nitroglycerin (Figure 3) in bovine coronary arteries [46]. Arteries were incubated with nitroglycerin for 2 h in the presence of GSH [46]. Under these conditions, nitroglycerin was readily degraded with a half-life of 26 min, with 1,3-dinitroglycerin being the predominant metabolite [46].

Conversely, co-incubation of the arteries with the GST inhibitors, sulphobromophthalein, and ethacrynic acid, decreased the rate of nitroglycerin degradation and formation of 1,3-dinitroglycerin [46]. Sulphobromophthalein and ethacrynic acid treatment resulted in a marked increase in the half-life of nitroglycerin from 26 to 66 min and 84 min, respectively, with a decrease in 1,3-dinitroglycerin generation [46]. The change in nitroglycerin degradation and 1,3-dinitroglycerin production suggested that in bovine coronary arteries, cytosolic GSTs are involved in vascular nitroglycerin metabolism [46].

It is notable that other GST inhibitors such as 6-(7-nitro-2, 1,3-benzoxadiazol-4-ylthio) hexanol (NBDHEX) and ezatiostat HCl (TLK199) have been extensively used in other studies and effectively suppress, primarily, GSTP1 [69,70]. However, sulphobromophthalein, ethacrynic acid, and basilen blue are more frequently used for inhibiting vasodilation [11,12,42,43,45,67,68]. This is because the latter inhibitors are more suited to inhibiting the interaction of GSTs with organic nitrates and also preferentially target the major GST involved in this biotransformation, namely GSTM1 [11,12,42,43,45,67,68]. 

## 6. Role of GST Isoform-Specific Biotransformation on Vasodilator Activity

The results above are supported by a later investigation that purified and characterized rat aortic GSTs and examined their role in the biotransformation of nitroglycerin [45]. The GST isoforms, GSTA (Ya and Yc), GSTM (Yb2), and GSTP (Yp), were detected in the rat aortic cytosol and purified using affinity chromatography and cation- and anion-exchange chromatography [45]. These studies demonstrated the GST Yc and GST Yb2/Yp isozymes could mediate nitroglycerin biotransformation [45].

Interestingly, degradation of nitroglycerin and GST activity was highly sensitive to the GSTM inhibitors, basilen blue and sulphobromophthalein [45]. Furthermore, significant inhibition of GST activity and nitroglycerin biotransformation was observed following the removal of the GSTM Yb2 isozyme from the rat aortic cytosol via immunoprecipitation [45]. This study indicated that GSTs are crucial in the de-nitration of nitroglycerin in rat aortic cytosol and that there was isoform-specific biotransformation by the GSTM Yb2 class [45]. Another study purifying GST isoforms from blood vessels identified five GST forms immunologically related to GSTM within the aorta and heart[12]. Furthermore, the activity of GSTM toward nitroglycerin was inhibited by GST inhibitors [12].

From the above data, it is evident that GSTs, particularly GSTM1, contribute to the biotransformation of nitroglycerin and organic nitrates to produce NO for vasorelaxation. This relationship of GSTs with NO demonstrates that they promote NO-mediated signaling. In contrast, other regulatory effects of GSTs exhibit inhibition of the activity of NO via their ability to directly bind and store NO as DNICs [6,8,15,21].

## 7. Conclusions and Future Directions

The proposed functions of GSTs have evolved from being solely involved in detoxification to more extensive roles in NO biology and vasodilation. Key observations are the requirement of GSH by GSTs to mediate the biotransformation of organic nitrates, such as nitroglycerin, to lead to NO generation [53]. This includes studies associating GST activity with vasodilation through this biotransformation mechanism [11,12,44,45,46,47,49,67,68]. Additionally, GSTs have multiple roles in NO metabolism that include the direct binding of DNICs for storage [8,9,15] and the interaction with the key NO-generating enzyme, iNOS, to promote its degradation [5]. As such, the functional role of GSTs are diverse and appear to bridge seemingly disparate biological processes.

Further studies examining the GSTs and their roles in regulating vasodilation via its interactions with NO could lead to new therapeutic avenues to treat hypertension and other related disorders. In particular, the *GSTM1* null genotype has been associated with an increased risk of blood pressure-related disorders such as preeclampsia and hypertension [71,72,73,74,75]. Investigations exploring the interaction of GSTM1 with NO, especially as DNICs, and the impact on sGC activation would provide novel insights for the treatment of these conditions and potentially advance the development of new vasodilators.

## Data Availability

Not applicable.
